# A Wrapper Feature Selection Algorithm: An Emotional Assessment Using Physiological Recordings from Wearable Sensors

**DOI:** 10.3390/s20010309

**Published:** 2020-01-06

**Authors:** Inma Mohino-Herranz, Roberto Gil-Pita, Joaquín García-Gómez, Manuel Rosa-Zurera, Fernando Seoane

**Affiliations:** 1 Department of Signal Theory and Communications, University of Alcalá, Alcalá de Henares, 28805 Madrid, Spain; roberto.gil@uah.es (R.G.-P.); joaquin.garciagomez@uah.es (J.G.-G.); manuel.rosa@uah.es (M.R.-Z.); 2 Institute for Clinical Science, Intervention and Technology, Karolinska Institutet, 17177 Solna Stockholm, Sweden; fernando.seoane@ki.se; 3 Department of Medical Care Technology, Karolinska University Hospital, 14157 Huddinge, Sweden; 4 Textile Materials Technology, Department of Textile Technology, Faculty of Textiles, Engineering and Businees Swedish School of Textiles, University of Boras, 50190 Boras, Sweden

**Keywords:** emotional assessment, physiological signal, feature selection

## Abstract

Assessing emotional state is an emerging application field boosting research activities on the topic of analysis of non-invasive biosignals to find effective markers to accurately determine the emotional state in real-time. Nowadays using wearable sensors, electrocardiogram and thoracic impedance measurements can be recorded, facilitating analyzing cardiac and respiratory functions directly and autonomic nervous system function indirectly. Such analysis allows distinguishing between different emotional states: neutral, sadness, and disgust. This work was specifically focused on the proposal of a k-fold approach for selecting features while training the classifier that reduces the loss of generalization. The performance of the proposed algorithm used as the selection criterion was compared to the commonly used standard error function. The proposed k-fold approach outperforms the conventional method with 4% hit success rate improvement, reaching an accuracy near to 78%. Moreover, the proposed selection criterion method allows the classifier to produce the best performance using a lower number of features at lower computational cost. A reduced number of features reduces the risk of overfitting while a lower computational cost contributes to implementing real-time systems using wearable electronics.

## 1. Introduction

In the literature, numerous studies show that the human body generates information regarding emotional state continuously. Often, people express their feelings verbally and through body gestures. However, emotional state and mental and physical load influence the individual at a physiological level, and such influences can be reflected in the parasympathetic activity of the autonomous nervous system (ANS). Its parts are the sympathetic nervous system (SNS) and the parasympathetic nervous system (PSNS). The main function of the SNS is to mobilize the body’s nervous system in what is known as the fight-or-flight response [1,2]. It is a physiological reaction produced by a perceived harmful event, attack, or threat to survival. The PSNS has its roots in the brain stern and spinal cord in the lower back. Its function is to bring back sanity after an emergency situation that led to the SNS taking control. Certain reactions are provided by SNS and PSNS in such organs as the eyes, heart, lungs, blood vessels, sweat glands, and digestive tract. When communicating human emotions, there are several tools involved, which humans use willfully and unconsciously to communicate their own emotions or feelings. Apart from gestures and speech, emotions modify some uncontrollable physiological signals produced in the human body; some can be read by the electrocardiogram (ECG), thoracic electrical bioimpedance (TEB), and skin conductance.

ECG signal analysis is widely used in medicine, since it offers information regarding diseases and disorders affecting the cardiac function. Several studies [3,4,5,6,7] conclude that emotions clearly affect the cardiac activity.

The TEB measurement is a non-invasive technique used to monitor the dynamics of the thoracic activity. This is directly related to the breathing. In the literature, it is possible to find multiples views about the relationship between emotions and breathing activity. A long time ago, in [8], the relationships between emotions and breathing and the possibility of controlling a anxiety and panic attacks through breathing were studied. Across many years it is possible to find numerous articles that study such relationship [9,10].

TEB has also been related to emotions in [11], wherein it was used to study the hemodynamic reactions to road traffic noise in young adults. The thoracic bioelectrical impedance is related to stress induced by traffic noise. Moreover, several studies have demonstrated that the analysis of ECG and TEB recordings can indeed provide information about the emotional status of individuals [12,13].

To register the red cited physiological signals, there are several types of devices. The proliferation of wearable sensing devices [14,15,16], such as smart watches and wrist and chest-bands, combined with the communication and data processing capabilities of smartphones, is boosting the potential for pervasive monitoring.

Emotional analysis with recorded ECG or TEB signals is understood as a classification problem, implemented with several steps: (a) meaningful features are calculated from the signals; (b) the vector of features is applied as input to a classifier, whose outputs indicate a person’s emotions. Meaningful feature selection is one the main tasks in classification for several reasons: (a) models can be simplified to make them easier to be interpreted; (b) the models can be trained in shorter times; (c) it helps to avoid the curse of dimensionality, enhancing generalization by reducing overfitting.

The main objective of this paper is to select the most valuable and efficient features to distinguish three emotional states—sadness, disgust, and neutral—using the physiological signals aforementioned.

The problems of generalization are given by several factors in emotional recognition. Generalization is a measure of how accurately an algorithm is able to classify previously unseen data. The capacity of generalization is related to the number of parameters to be tuned during training and the number of samples available for training the classifier. Generalization error can be minimized by avoiding overfitting in the learning algorithm. A common strategy to reduce the number of parameters to be tuned, so as to reduce the possibility of overfitting, is feature selection. It allows reducing the dimension of the input vector, and consequently, the complexity of the system.

In the literature, numerous algorithms to improve generalization can be found; for example, using neural networks [17] or combining genetic algorithms (GA) with the hybrid learning algorithm (HLA) [18]. A study of generalization capabilities of SVMs trained with k-fold cross validation was carried out in [19].

In this paper, an algorithm is proposed whose main objective is to improve the selection of features to minimize these undesirable generalization problems. We compared two different fitting functions aimed to reduce the loss of generalization: one of them is based on the use of a standard error function as the selection criterion and the other is the proposed k-fold-based fitting function.

## 2. System Overview

### 2.1. System Architecture

**Figure 1:**
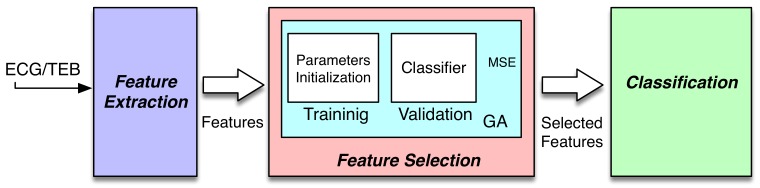
Complete system.

A functional work-flow overview of the system is depicted in Figure 1. The system can be included in the known passive brain-computer interface (pBCI). These systems register biosignals for assessment of emotional and mental workload. In [20], a review is presented about the pBCI with the current trends and future directions. These systems are the interface between humans and computers through signal acquisition, feature extraction, and finally, feature selection.

The first block represents the feature extraction task, where the features used in this study were calculated in order to extract the information from biosignals. The second block, the feature selection block, aims at selecting the most efficient features, which in this paper was performed using an evolutionary algorithm and a quadratic classifier. Finally, the classification block—where the classifier produces outputs to classify the input signals minimizing an objective function, such as the mean squared error. The classification task is implemented in both the feature selection block and the classification block. In both cases, the classifier selected was a quadratic classifier; concretely, the least square diagonal quadratic classifier (LSDQC), which is explained in detail in [21].

**Figure 2:**
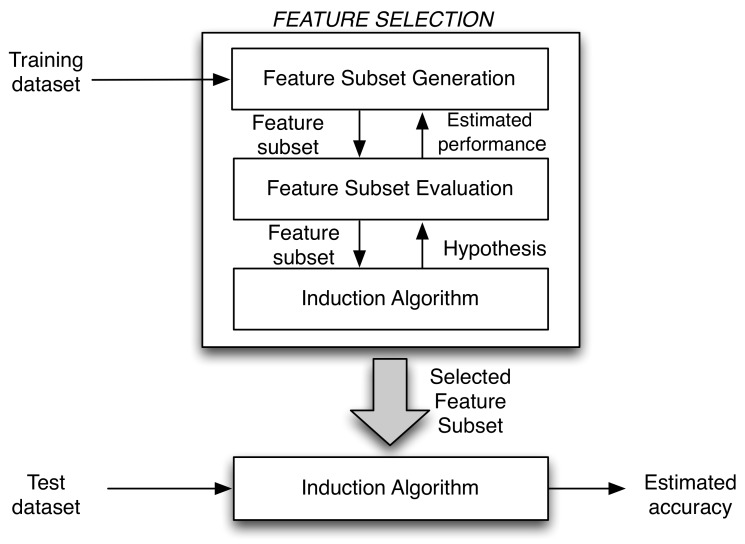
Wrapper approach scheme.

The Feature Selection block was based on wrapper methods, which were proposed in [22]. These models require a predetermined learning algorithm to select features and use their performance to evaluate and determine which features are selected. Usually, wrapper methods can generate potential feature subsets with high accuracy because of the good matching of that subset of features with the learning algorithms. The disadvantage comes from the large computational cost, due to the use of the classifier for each subset of features. The concept of wrapper approach is presented in Figure 2. A deeply studied variant of the wrapper model is the randomized one, which relies on search strategies such as genetic algorithms (GAs) [23], hill climbing [24], and simulated annealing [25]. In some cases, part of the classifier design process (tuning of some parameters) is embedded within feature selection in the optimization process [26].

The training and validation processes are carried out offline. Once the best features are obtained, which are selected based on performance and computational cost, the real-time system only calculates the features that were selected. With these features and the optimized model, it is possible to assign the pattern to a class.

### 2.2. Feature Extraction

The ECG and TEB signals are analyzed to generate several characterization features, such as time analysis, QRS complex analysis or heart rate variability (HRV) [27,28], and the power ratios in different frequency bands [29,30].

**Figure 3:**
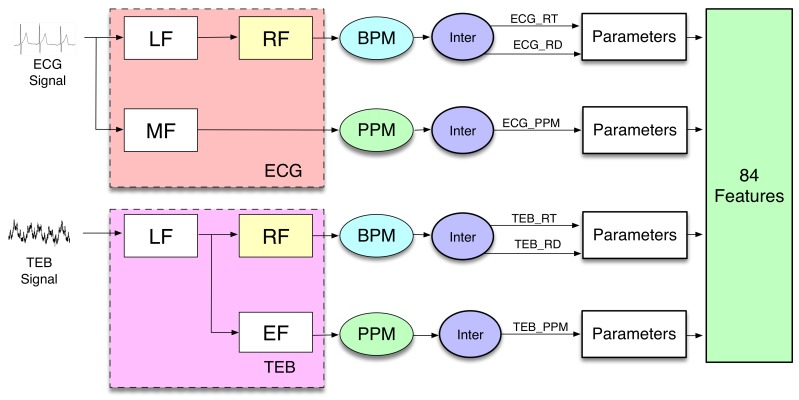
Feature extraction scheme.

In order to include information about time variation, several temporal averages of calculated features are included in the features set. In the present work, the features are calculated similarly to [31], as it is shown in Figure 3. The scheme shows a block diagram, where it is possible to distinguish the different functional operations implemented on the biosignal recordings to extract the features.

In the present work, the features were determined every 10 s. The choice of this value is a compromise between the minimum time to calculate the features and the implementation in real time. Since the feature extraction is expected to be implemented in a smartphone, it is necessary to calculate the computational complexity associated with each feature.

The blocks Low Frequency (LF), Respiration Frequency (RF), Medium Frequency (MF), and Electrocardiogram Frequency (EF) are filters.

LF and MF are anti-aliasing filters, which allow the use of interpolated finite impulse response (IFIR) filters [32]. The outputs of these anti-aliasing filters are applied to two different IFIR filters, RF and EF, with a stretch factor SF = 25 for the ECG measurement and SF = 10 for the TEB measurement.

For calculating the computational cost for each filter, we used the following reasoning: The cost of the anti-aliasing filter was obtained as $C_{ANT}=N\cdot F$, N being the filter order and F the sampling frequency of the measurement used (ECG or TEB). Analogously, the cost associated with the IFIR filter is calculated with $C_{IFIR}=\frac{N\cdot F}{SF}$.

In Table 1 it is possible to observe the kind of filters used, the cut-off frequencies, the order, and computational cost.

The outputs of the filters are applied to different blocks denominated BPM (breaths per minute) and PPM (pulsations per minute). The block BPM considers the signal to be sine-shaped, and it periodically determines the minimum and the maximum values of the signal, allowing estimation of the number of breaths per minute and its amplitude.

The block PPM obtains the pulsations per minute. The outputs of BPM and PPM blocks are used by the next block, denominated Inter, to generate interpolated signals, resulting from piece-wise constant interpolation to the last known values of the number of breaths per minute, its amplitude, or the number of beats per minute. The sampling frequency of the generated signals after interpolation is 50 Hz. The associated computational cost analysis is included in Section 4.3.

The block parameters represent the calculations of 14 statistical parameters (statistics), which are: trimmed mean of 25%, median, percentile 25%, percentile 75%, kurtosis, skewness, standard deviation, mean absolute deviation, mean absolute deviation, geometric mean, harmonic mean, baseline, maximum, minimum, and mean. Finally, we obtain three signals from each measurement. Since the number of parameters used is 14, the total number of features is 84, 42 from each measurement. The computational cost calculated for these blocks is shown in the results section.

### 2.3. Emotion Classifier

In feature selection using GA, the classifier used to test the performance with the selected features at each stage must be simple for the algorithm to be feasible. The simplest classifier is a linear classifier, but it only implements linear boundaries to separate the classes. In this study, we relied on the least square diagonal quadratic classifier [21], which can be used to implement closed boundaries, and at the same time is quite simple.

Let us consider a set of training patterns $x={\left[x_{1},x_{2},\ldots ,x_{L}\right]}^{T}$, where each of these patterns is assigned to one of the possible classes denoted as $C_{m}$m=1,…,M., *M* being the number of classes. In a quadratic classifier, the decision rule can be obtained using a set of k combinations, as shown in Equation (1).

(1) $$y_{m}=w_{m0}+\sum_{n=1}^{L}w_{mn}x_{n}+\sum_{n=1}^{L}v_{nm}x_{n}^{2}.$$

The pattern matrix Q, which contains the input features for classification and their quadratic values, is expressed in Equation (2).

(2) $$Q=\left[\begin{array}{ccccc} 1 & 1 & 1 & \ldots & 1\\ x_{11} & x_{12} & x_{13} & \ldots & x_{1N}\\ \vdots & \vdots & \vdots & \ddots & \vdots \\ x_{L1} & x_{L2} & x_{L3} & \ldots & x_{LN}\\ x_{11}^{2} & x_{12}^{2} & x_{13}^{2} & \ldots & x_{1N}^{2}\\ \vdots & \vdots & \vdots & \ddots & \vdots \\ x_{L1}^{2} & x_{L2}^{2} & x_{L3}^{2} & \ldots & x_{LN}^{2} \end{array}\right].$$

Defining V, the weights matrix, with Equation (3).

(3) $$V=\left[\begin{array}{ccccccc} w_{10} & w_{11} & \ldots & w_{1L} & v_{11} & \ldots & v_{1L}\\ \vdots & \vdots & \vdots & \vdots & \vdots & \vdots & \vdots \\ w_{M0} & w_{M1} & \ldots & w_{ML} & v_{M1} & \ldots & v_{ML} \end{array}\right].$$

The output of the quadratic classifier is obtained in Equation (4).

(4) Y=V·Q.

The target matrix, which contains the labels of each patterns is defined as:

(5) $$T=\left[\begin{array}{ccccc} t_{11} & t_{12} & t_{13} & \ldots & t_{1N}\\ \vdots & \vdots & \vdots & \ddots & \vdots \\ t_{M1} & t_{M2} & t_{M3} & \ldots & t_{MN} \end{array}\right],$$

where *N* is the number of data samples, and $t_{mn}=1$ if the *n*-th pattern belongs to class $C_{m}$, and 0 otherwise. Then, the error is the difference between the outputs of the classifier and the correct values, which are contained in the target vector:

Consequently, the mean squared error is defined with Equation (6).

(6) $$MSE=\frac{1}{N}{∥Y-T∥}^{2}=\frac{1}{N}{∥V\cdot Q-T∥}^{2}.$$

In the least squares approach, the weights are adjusted in order to minimize the mean squared value of this error (MSE). The matrix V which minimizes Equation (6) is obtained with the Wiener–Hopf equation:

(7) $$V=T\cdot Q^{T}\cdot {\left(Q\cdot Q^{T}\right)}^{-1}.$$

This expression allows us to determine the values of the coefficients that minimize the mean squared error for a given set of features.

To avoid the loss of generalization in the results while maximizing the accuracy in the estimation of the error rate, k-fold cross-validation was used in the experiments, with *k* set equal to *S*, the number of subjects available in the design database. Thus, the data were divided into *k* folds or subsets containing data from each subject, and each time, the registers from one given subject were used as a test set, with the data from the k−1 subject used for the training task.

Therefore, ${Q|}_{k=s}$ stands for the sub-matrix of Q containing the data of the *s*-th fold; that is, data from the *s*-th subject of the database. Thus, in the *k*-fold validation process, the output of the classifier for the *s*-th individual is obtained using data from the remaining individuals; that is, ${Q|}_{k\neq s}$. Thus, taking into account Equations (4) and (7), we obtain the output of the classifier for the *s*-th subject of the database as follows:

(8) $${Y|}_{k=s}{=V|}_{k\neq s}{\cdot Q|}_{k=s}{=T|}_{k\neq s}{Q|}_{k\neq s}^{T}{\left(Q\right|}_{k\neq s}{\cdot Q|}_{k\neq s}^{T}{{)}^{-1}\cdot Q|}_{k=s}.$$

Once this output is obtained, the classification decision is made, determining which term of the output is maximum. The classification error rate is then estimated as the average of the k-folds classification errors.

## 3. Proposed Feature Selection Method

For the feature selection task, the algorithm selected was a tailored version of a genetic algorithm (GA) [33]. The performance of GAs is based on the application of evolutionary laws (crossover, mutation, and selection of the fittest or elitism) to an initial generic population of possible solutions to meta-heuristically find a good solution to the problem. With these algorithms, a set of features can be found out as the solution of an optimization problem with the objective of minimizing the classification error with a constraint related to the number of operations, $N_{op}$. Since the application of GA requires the calculation of the classifier error for each combination of features in the current population of feature sets, the classifier should not be complex in order to reduce the computational complexity of the solution. For this reason, quadratic classifiers were used; they combine reduced computational complexity and reasonable performance.

To understand the procedure used, it is important to note that to determine the set of features, we took into account both the performance of the classifier (in terms of error rate) and the computational complexity. For simplicity, we considered the number of simple operations per second ($N_{op}$) as an indicator of the computational complexity of the real-time implementation of a given set of features, since this value is proportional to the CPU load of the final implementation.

As it was stated above, the selection of the fitting function in the GA-based feature selection process is a critical issue, since its choice may cause generalization problems, due to the small number of available data. To avoid generalization problems risen from the limited size of the dataset, in this paper, two fitting functions were compared: a standard MSE-based function, and a novel k-fold-based fitting function, which aims at increasing the generalization capability of the feature selection process.

### 3.1. Standard Design Error Optimization (SDEO)

In a first approach, we select the set of features that minimize the mean squared error over the design set. At this point we have to consider that, as it was stated above, we use a *k*-fold validation using the different individuals of the database as folds (*k* must match *S*, the number of individuals in the database). In this validation method, the database is divided into *k* folds, one for each individual in the dataset. The design set for the *s*-th iteration of the *k*-fold validation process is denominated, at this point, ${Q|}_{k\neq s}$ (since it contains all data except those patterns included in the *s*-th fold), and the test set for the *s*-th iteration of the *k*-fold validation process is denominated ${Q|}_{k=s}$ (containing only those patterns included in the *s*-th fold). The MSE of the k-fold validation process can be calculated using Equation (9).

(9) $$MSE=\frac{1}{S}\sum_{s=1}^{S}\frac{1}{N_{s}}{∥V|}_{k\neq s}{\cdot Q|}_{k\neq s}-T{{|}_{k=s}∥}^{2}.$$

It is worth mentioning that feature selection is part of training; therefore, in order to guarantee generalization of the results, the test set cannot be used in order to select the best features. Thus, the SDEO for the *s*-th iteration of the *k*-fold validation process is exclusively obtained using the design data of that iteration; that is, the mean squared error over the design set is expressed using Equation (10).

(10) $$SDEO_{s}=\frac{1}{\sum_{i\neq s}N_{i}}{∥V|}_{k\neq s}{\cdot Q|}_{k\neq s}-T{{|}_{k\neq s}∥}^{2}.$$

### 3.2. *k*-Fold-Based Error Optimization (KFBEO)

Taking into account that the database does not contain a large number of data from a large number of individuals, the SDEO might present loss of generalization in the selection of the features. That is, the features selected in each iteration of the *k*-fold process may be especially good for the given design set. In order to overcome generalization loss, we propose the use of an additional k-fold optimization process for feature selection. In the design test, to find out the select features, the output is obtained for systems trained with the folds in the design set, but one is left out which has input equal to that fold that was left out. The KFBEO proposed to be minimized in order to determine the selected features for the *s*-th iteration of the *k*-fold validation process will be consequently given by Equation (11), by averaging over the S−1 folds (being *S* the number of subjects in the database).

(11) $$KFBEO_{s}=\frac{1}{\sum_{\begin{array}{c} n=1\\ n\neq s \end{array}}^{S}N_{n}}\sum_{\begin{array}{c} n=1\\ n\neq s \end{array}}^{S}{\left||V\right|}_{k\neq (n,s)}\cdot Q{{|}_{k=n}-T|}_{k=n}|{|}^{2},$$

where ${V|}_{k\neq (n,s)}$ are the weights of the quadratic classifier, determined using Equation (7) over the database excluding both the *s*-th subject (the test subject of the main *k*-fold validation process), and the *n*-th subject (the design subject of the *k*-fold process related to the evaluation of the KFBEO fitting function).

## 4. Experiments and Results

### 4.1. Database Description

The database consists of the ECG and TEB signals recorded from 40 subjects, while the subjects watched certain sequences of films whose objective was to elicit the different emotions under study, neutral emotion, sadness, and disgust. The sample frequency was 100 Hz for TEB and 250 Hz for ECG. The database is described in detail in [31] and it can be downloaded from https://www.mdpi.com/1424-8220/19/24/5524 associated with [34]. A brief description of the database can be found in Appendix A.

### 4.2. Parameters Used for Evaluation

To quantify the error and improvement obtained using the proposed algorithm, several parameters are used:

- Computational cost.
- Performance of the classifier measured with the MSE. This error can be calculated when features are selected using any one of the proposed methods. In the present case, the database is balanced, which means that the number of patterns of each class is the same. For this reason, it is enough to analyze the accuracy, since there is no possibility to fall into the accuracy paradox [35].
- Ranking of features. It is very important to know which features provide more valuable information so as to reduce calculation time and computational cost.
- Power consumption in a specific smartphone. The system has been implemented in a smartphone, in order to compare the required power consumption with each possible solution, when it is implemented in a embedded device.

### 4.3. Results

#### 4.3.1. Computational Cost

The computational cost obtained for the execution of algorithms and the extraction of features is reported as follows. Table 2 lists the computational cost obtained for each of the implemented filters (LF, MF, RF, and EF), while for the algorithms PPM and BPM the estimated computational costs are 9050 and 8800 operations per second, respectively (the explanation about the way to estimate the computational cost is part of Section 2.2).

Some of the features are obtained by calculating statistics over some primary features. The number of operations required to calculate these statistics must also be considered to evaluate the complexity of our solution. The computational cost for the calculation of these statistics is shown in Table 3.

#### 4.3.2. Classification Performance

**Figure 4:**
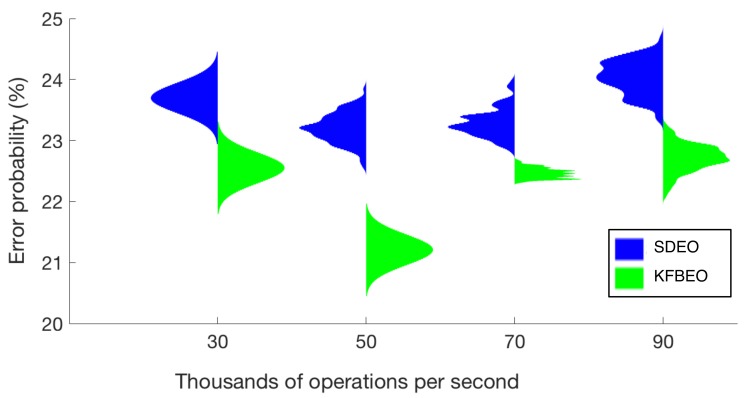
Error probabilities obtained using standard design error optimization (SDEO) and fold-based error optimization (KFBEO).

Figure 4 contains the violin plots for the classification performance obtained by each of the algorithms. The obtained classification error is plotted in function of the computational cost expressed as the maximum number of operations per second required using the features selected by each of the algorithms. The classification errors are presented as means of 100 estimations, and for the sake of clarity, they were normalized for the figure (the most valuable information is the mean value and how the results spread around the mean value). The minimum error probability obtained using the SDEO is approximately 26.5% (mean value), for 50,000 operations per second, using 35 features. The minimum mean error probability with the proposed KFBEO method for feature selection is around 22.5% for 50,000 operations per second with 10 features.

#### 4.3.3. Ranking of Selected Features

The label of each feature was constructed from the name of the biosignal and the algorithm used: ECG_PPM, ECG_RD, ECG_RT, TEB_BPM, TEB_RD, and TEB_RT. Said names are followed by the names of the parameter or statistic used, respectively. Feature selection has been repeated 100 times, being 40-fold. The number of attempts to estimate the percentage of positive feature selections is the product of k and the number of the repetitions (100).

Table 4 presents the features that have been selected in more than 20% of the experiments. In this case, 60 of the 84 features were selected at least once.

The number of features selected at least once using the KFBEO is 36. And number of features selected more than 20% of the attempts is five; they are shown in Table 5.

The number of features selected for obtaining the best result for the SDEO is 35 features, meanwhile for the KFBEO, that number is 10 features. That is the reason for which the computational cost of KFBEO is lower than the computational cost of SDEO.

#### 4.3.4. Power Consumption

Feature extraction was implemented in a Galaxy Pocket smartphone, manufactured by Samsung Electronics, Co., running with an 832 MHz CPU, 512 MB, and a 1200 mAh battery. In order to assess whether the system was suitable for real-time applications using the given smartphone, the application was executed for four hours of full performance, during which the battery level decreased an additional 10% when compared with the standard battery usage with the smartphone operating in sleep mode. The battery consumption was mainly due to Bluetooth. Regarding resource management, the app running the feature extraction used less than 6% of the CPU resources.

## 5. Discussion

Nowadays, the analysis of biological signals to determine the emotional state in real-time is a promising research field [36,37,38]. Through the analysis of electrocardiogram and thoracic impedance recordings obtained with wearable sensors to monitor cardiac and breathing activity, respectively, it is possible to distinguish among three different emotions: neutral emotion, sadness, and disgust. In the study of the ECG and TEB signals, numerous characterizing features can be obtained, carrying information useful for different purposes. Working with a large number of features in classification applications might impact in the generalization ability of the obtained classifiers, resulting in systems with sub-optimal performances when applied in real scenarios [39].

The classifier which uses the features selected with the k-fold-based approach achieves a lower probability of error than the classifier which uses the features selected with the standard fitting function. This is due to an improvement in generalization during training, because the database for training does not contain a large amount of data from many individuals. The use of the SDEO produces loss of generalization.

In this work, we aimed at selecting the best features targeting both low error rate and reduced computational complexity. A wrapper feature selection algorithm based on GA has been used [40,41]. In these cases, the selection of the fitting function in the GA-based wrapper feature selection process is of paramount importance, since a wrong choice may lead to generalization issues. In cases with a small amount of data available, the selection of the fitting function becomes critical.

As an alternative approach, we propose applying a k-fold technique to the evaluation of the fitting function, each fold corresponding to one subject of the design set. The results show that the KFBEO renders a lower error probability than the SDEO. The KFBEO renders an improvement, which is around 3% in error probability using the same number of operations per second. Moreover, the error obtained is in all cases lower using the KFBEO.

Besides, the number of features selected for obtaining the best result for the SDEO is near to 35, but for the proposed KFBEO is only 10 features. The number of features is likely to affect the performance of the classifier, since increasing the number of features also increases the possibility of over-fitting [42]. Concerning the operations per second required to obtain the best result, the number is remarkably lower for KFBEO than SDEO, which implies that the computational cost is smaller with KFBEO than with SDEO.

## 6. Conclusions

Comparing the results rendered by the emotions classification system using the features selected with SDEO an KFBEO, we can conclude that the error probability provided by the classifier, which uses the features selected with the KFBEO, is notably better than the error probability provided by the classifier, which uses the features selected with SDEO. In addition, as the number of features selected with KFBEO-based approach is lower, the resulting computational complexity is lower too.

Comparing classifiers with a similar number of operations fed with features selected with both methods, an improvement of 3% in error classification is obtained.

According to the number of features needed for obtaining the best results in each algorithm, the SDEO selects 35 features; meanwhile, the KFBEO only requires the selection of 10. This implies that the classifier trained with the KFBEO presents a higher tolerance to overfitting, since less features are used than when using SDEO fitting function for feature selection. The number of features selected is related to the computational cost, and therefore, to the battery consumption. The implementation of the proposed classification solution in a smartphone is feasible, since the computational cost is low in comparison with the number of operations per second that a smartphone can perform. Therefore, a future work can be to test the features in other realistic mobile computing environments with Android or i-OS.

## Figures and Tables

**Table 1:** Types of filters and computational cost.

Name	Type	Order (N)	Cutoff (Hz)
$LF_{ECG}$	Low-pass (anti-aliasing)	100	3
$MF_{ECG}$	Low-pass (anti-aliasing)	100	30
$RF_{ECG}$	Band-pass (IFIR)	1150	0.1–0.5
$LF_{TEB}$	Low-pass (anti-aliasing)	100	3
$RF_{TEB}$	Band-pass (IFIR)	400	0.1–0.5
$EF_{TEB}$	Band-pass (IFIR)	400	0.8–2.9

**Table 2:** Computational cost for each filter in the real-time implementation.

Name	${\mathrm{LF}}_{\mathrm{ECG}}$	${\mathrm{MF}}_{\mathrm{ECG}}$	${\mathrm{RF}}_{\mathrm{ECG}}$	${\mathrm{LF}}_{\mathrm{TEB}}$	${\mathrm{RF}}_{\mathrm{TEB}}$	${\mathrm{EF}}_{\mathrm{TEB}}$
$Cost(N_{op})$	25,000	25,000	11,500	10,000	4000	4000

**Table 3:** List of statistics and number of operations per second required.

Parameter	Operations per Second
Trimmed mean of 25%	27,805
Median	27,580
Percentile 25%	27,580
Percentile 75%	27,580
Kurtosis	2701
Skewness	2101
Standard deviation	1201
Mean absolute deviation	1200
Geometric mean	3901
Harmonic mean	3301
Baseline	550
Maximum	300
Minimum	300
Mean	300

**Table 4:** Features selected more than 20% in all experiments for SDEO.

Feature	Percentage (%)
TEB_PPM_Baseline	100
TEB_PPM_Max	99.9
TEB_PPM_Mean	99.9
TEB_PPM_Min	99.3
TEB_PPM_Std	97.6
TEB_PPM_Mad	97.6
TEB_PPM_Harmean	77.3
TEB_RT_Baseline	73.3
TEB_RT_Mean	73.3
TEB_RT_Min	72.8
TEB_RT_Max	44.3
E_PPM_Mean	44.3
E_PPM_Min	44.2
E_PPM_Baseline	43.9
TEB_PPM_Skewness	43.6
E_PPM_Max	42.7
E_PPM_Std	40.5
E_PPM_Mad	39.7
TEB_RD_Baseline	30.0
TEB_RD_Mean	30.0
E_PPM_Harmean	27.9
TEB_RT_Mad	25.1
TEB_RD_Mad	25.1
TEB_RD_Max	20.6

**Table 5:** Features selected more than 20% in all experiments for KFBEO.

Feature	Percentage (%)
TEB_PPM_Baseline	100
TEB_RT_Max	100
TEB_PPM_Mean	97
E_PPM_Mad	48.73
E_PPM_Mean	48.58

**Figure A1:**
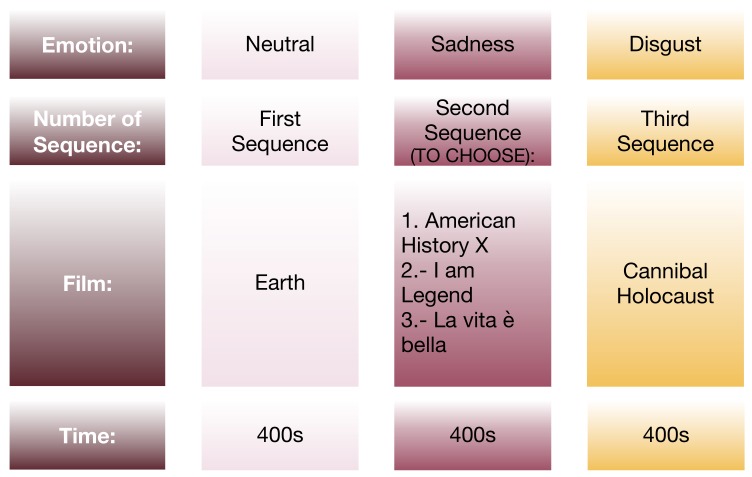
Films used to elicit the three emotions: neutral, sadness and disgust.

## Appendix A. Database

The description of the database in detail can be found in [31] and https://www.mdpi.com/1424-8220/19/24/5524 associated with [34]. The database consists of the ECG and TEB signals recorded from 40 subjects (students and climbers) aged between 20 and 49 years, of which 12 were females and 28 males. The sampling frequency is 100 Hz for TEB and 250 Hz for ECG. The total experiment lasts approximately 90 min. All of the experiments were performed under the conditions of respect for individual rights and ethical principles that govern biomedical research involving humans, and written informed consent was obtained from all participants. The experiments were approved by the Research Ethics Committee at the University of Alcala, and the protocol number assigned to this experiment is CEI: 2013/025/201130624.

The part used in the present paper studied the emotional response, in which the main objective is to distinguish among three different emotions:

- Neutral emotional response
- Sadness emotion
- Disgust emotion

For this purpose, the subject under study watched three sequences of several films. In Figure A1 it is possible to observe the films used to elicit the three emotions under study. The sequences selected were watched twice, and we used the recordings from the last 200 s of each movie, generating 400 s for the neutral emotion, 400 s for sadness and 400 s for disgust for each subject. The first film was *Earth*, the second one was elected by the user among *American History X*, *I am a legend*, or *La vita é bella* and finally *Cannibal Holocaust*.
